# A Case of Sarcoidosis Masquerading as Right Middle Lobe Consolidation

**DOI:** 10.7759/cureus.28590

**Published:** 2022-08-30

**Authors:** Joshua L Aron, Nathan Douthit, Tonya Bradley

**Affiliations:** 1 Rheumatology/Dermatology, Edward Via College of Osteopathic Medicine, Auburn, USA; 2 Discipline Chair for Internal Medicine, Edward Via College of Osteopathic Medicine, Auburn, USA; 3 Family Medicine, Auburn Pediatric and Adult Medicine, Auburn, USA

**Keywords:** pulmonary consolidation, acute sarcoidosis, atypical presentation of sarcoidosis, high-resolution ct scan, pulmonary sarcoidosis

## Abstract

Sarcoidosis is a multisystem granulomatous disorder that is characterized histologically by noncaseating granulomas. Typically, it presents clinically in young adults with initially one or more of the following: hilar lymphadenopathy, pulmonary reticular opacities, and skin, joint, and/or eye lesion. Radiographic findings commonly include bilateral hilar and mediastinal lymphadenopathy and pulmonary reticular opacities but may resemble pneumonia with airspace consolidation and opacities. We report a case of sarcoidosis that presented as a persistent case of pneumonia. This case is a reminder that common diseases such as pneumonia are not always what they seem, and diseases such as sarcoidosis that have specific characteristics may not present traditionally each time.

## Introduction

Sarcoidosis is a multisystem granulomatous disorder that typically presents in adults between the ages of 20 and 60 [1]. It is characterized by clinical and pathological findings that include bilateral hilar adenopathy; pulmonary reticular opacities; skin, joint, and/or eye lesions; and noncaseating granulomas on histology [1]. In addition, commonly associated laboratory abnormalities include leukopenia and elevated erythrocyte sedimentation rate (ESR), C-reactive protein (CRP), and angiotensin-converting enzyme (ACE) levels [1]. Here, we present the case of a patient who developed a complicated case of right middle lobe consolidation and chronic cough even after the resolution of pneumonia symptoms. While in the hospital, he had elevated CRP and D-dimer. His chest X-ray and computed tomography angiography (CTA) revealed bilateral hilar and mediastinal lymphadenopathy; however, he had no other clinical or laboratory findings indicative of sarcoidosis. Further evaluation of his sarcoid was only indicated when workup for pneumonia was indeterminate and his cough and bilateral hilar lymphadenopathy were found to be persistent after discharge from the hospital. Therefore, we present a case of sarcoidosis presenting as a complicated case of pneumonia in the absence of many characteristic findings of sarcoidosis.

## Case presentation

**Figure 1:**
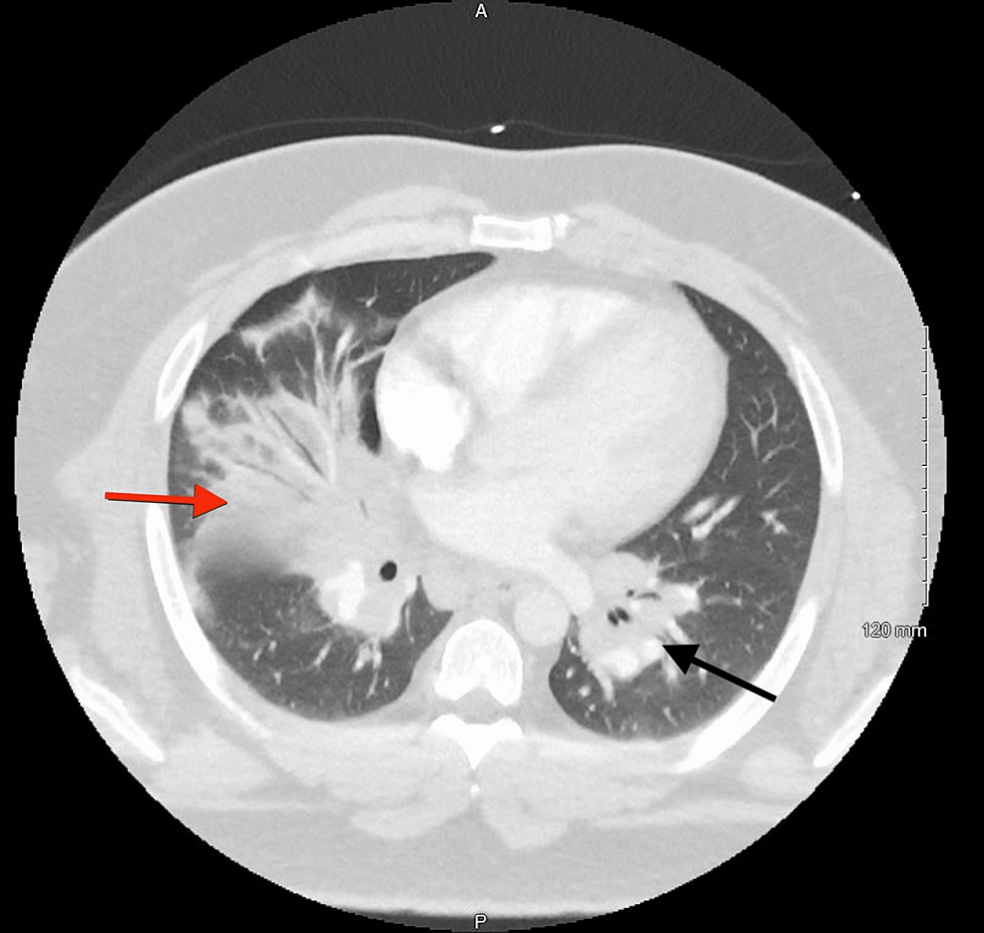
Mediastinal and bilateral hilar lymphadenopathy with right middle lobe airspace consolidation and pneumonia. Black arrow: hilar lymphadenopathy. Red arrow: airspace consolidation.

**Figure 2:**
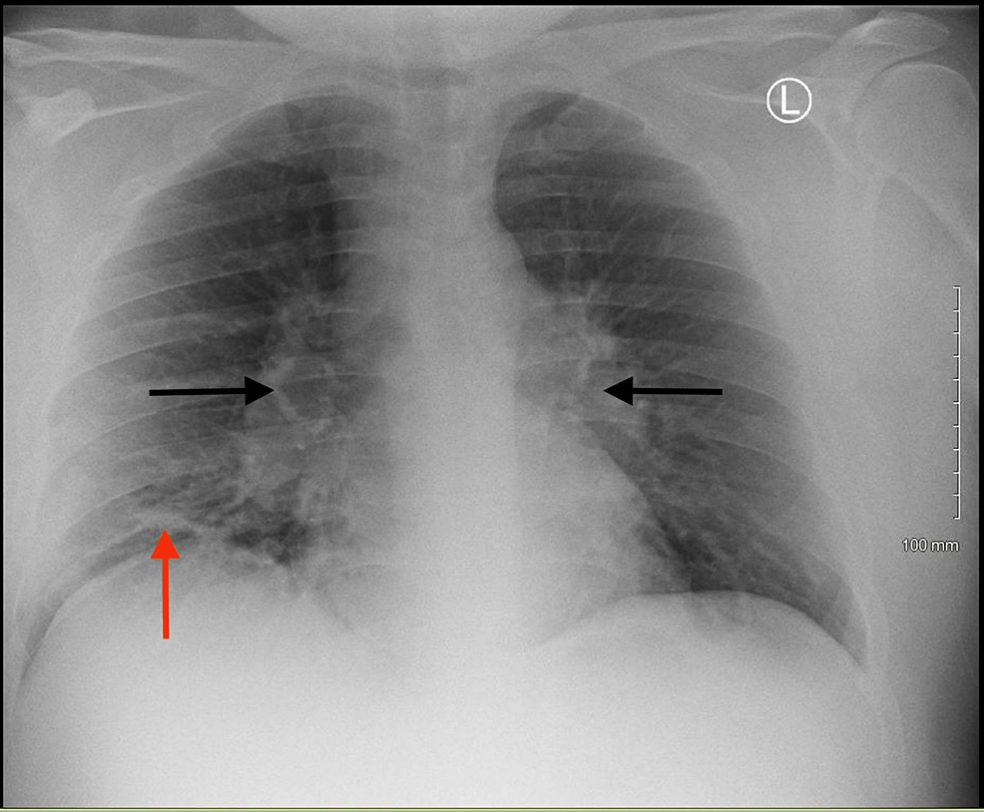
Mediastinal and hilar lymphadenopathy with airspace consolidation of the right middle lobe. Black arrows: hilar lymphadenopathy. Red arrow: airspace consolidation.

**Figure 3:**
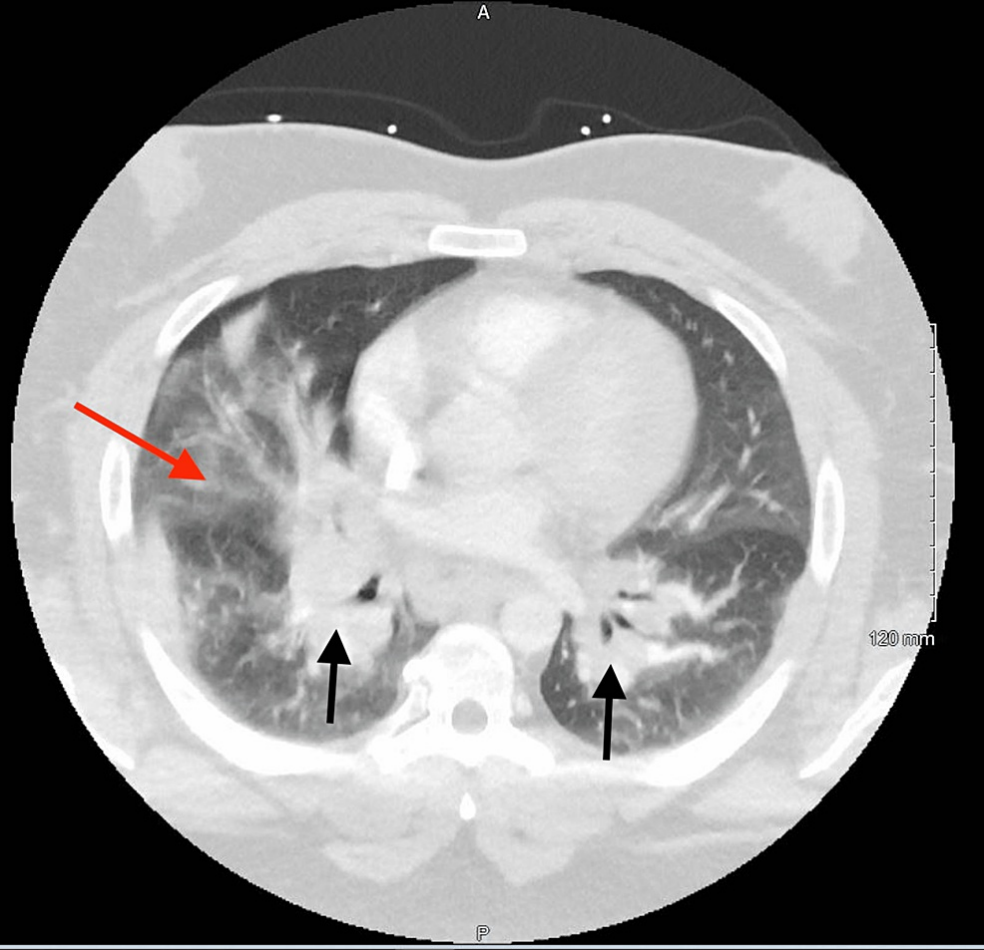
Prominent mediastinal and hilar lymphadenopathy with a partial clearing of the right lower lobe consolidation and pneumonia. Black arrows: hilar lymphadenopathy. Red arrow: airspace consolidation.

A 30-year-old white male with a medical history significant for lower extremity cellulitis, gout, childhood asthma, obstructive sleep apnea, and obesity presented with sepsis secondary to possible pneumonia. He had experienced 14 days of worsening cough, fever, and shortness of breath. The patient was initially seen by his primary care provider (PCP) 13 days before admission. He was diagnosed with a flare of childhood asthma and was given oral steroids and an albuterol inhaler. He presented to the Emergency Department (ED) eight days prior to admission with worsening symptoms. At the ED a CTA was performed that showed mediastinal and bilateral hilar adenopathy consistent with sarcoidosis or lymphoma and right middle lobe consolidation consistent with pneumonia (Figure 1). He was given a one-time intravenous dose of 750 mg of levofloxacin and discharged. On the day of the admission, he presented again to the ED with continued pneumonia-like symptoms. After a thorough evaluation, he was admitted to the inpatient service for sepsis and worsening pneumonia, and he was started on intravenous vancomycin 1,500 mg and intravenous piperacillin-tazobactam 4.5 g. His medications before admission included oral allopurinol 300 mg once per day, inhaled albuterol one puff every six hours, budesonide/formoterol 160 µg-4.5 µg inhaler one puff twice per day, and oral montelukast 10 mg once per day. On examination, vitals were significant for a heart rate of 144 beats per minute, respiratory rate of 24 breaths per minute, blood pressure of 131/68 mmHg, a temperature of 103°F, SaO_2_ of 93% on room air, and body mass index of 47. Laboratory examination was significant for a white blood cell count of 9.3 × 10^3^/µL and an elevated CRP of 5.9 mg/dL and a D-dimer of 4.21 mg/L. Repeat chest X-ray and CTA showed continued mediastinal and hilar lymphadenopathy with the consolidation of the right middle lobe and no evidence of pulmonary emboli (Figures 2, 3). The patient underwent a bronchoscopy on day three of admission. Other significant diagnostic findings included a down-trending CRP level throughout the hospital course, negative blood cultures, normal ACE and antinuclear antibody (ANA) levels, negative Biofire respiratory panel, and indeterminate bronchoscopy with negative bronchoalveolar washing (BAL) cultures.

Over the course of a few days, the patient’s shortness of breath slowly improved with a continued course of empiric antibiotics, and he was discharged from the hospital to follow up with his PCP and pulmonologist. Upon follow-up, he continued to have a chronic mild cough and new onset of erythematous nodules on his bilateral anterior lower legs. Pulmonary function testing was completed and was unremarkable. A positron emission tomography (PET) scan was performed, and the findings included extensive lymphadenopathy which was suspicious for sarcoidosis. Follow-up CTA was also performed and showed unchanged hilar and mediastinal lymphadenopathy with resolved right middle lobe consolidation. A decision was made to biopsy the lesions with a mediastinoscopy, and pathology revealed non-necrotizing granulomatous inflammation for a final diagnosis of sarcoidosis.

## Discussion

Sarcoidosis is a multisystem granulomatous disorder that typically presents in young adults between the ages of 20 and 60 [1]. Sarcoidosis is two to three times more common in black Americans than white Americans and twice as common in females, with the highest prevalence being among black American females [2]. The etiology is unknown, and its prevalence is estimated at 50 to 60 per 100,000 population worldwide [1,2].

Sarcoidosis tends to have a distinct clinical presentation that includes initially one or more of the following findings: bilateral hilar adenopathy, pulmonary reticular opacities, and skin, joint, and/or eye lesions. The disease most frequently involves the lung but has also been shown to involve a range of organs, including, but not limited to, the skin, lymph nodes, eyes, liver, muscle, kidneys, nervous system, heart, and kidneys. When the disease involves the lungs, it can commonly present with cough, dyspnea, chest pain, fatigue, weight loss, malaise, and fever. Furthermore, because it can involve so many different organ systems, patients may also have symptoms such as visual changes, skin lesions such as erythema nodosum, dry eyes or mouth, palpitations, joint pain, muscle weakness, parotid swelling, and syncope [1].

Pulmonological radiographic findings for sarcoidosis can be classified by typical and atypical characteristics [3-5]. Imaging techniques such as radiography, CT, PET, and endobronchial ultrasonography can be useful in the evaluation and diagnosis of sarcoidosis [1,3]. Chest radiography is typically the initial evaluation tool, but in recent times, high-resolution computed tomography (HRCT) has become the superior method of evaluating pulmonary lesions [3]. Typical HRCT pulmonary findings of sarcoidosis include hilar and mediastinal lymphadenopathy, nodules, reticular opacities, and bronchiectasis [3,5]. Atypical findings include unilateral lymphadenopathy, ground-glass opacities, interlobar septal thickening and linear opacities, pleural effusions, and airspace consolidation and opacities [3,5].

When airspace consolidation and opacities are seen in sarcoidosis, it is called alveolar sarcoid [3-5]. Alveolar sarcoid has been thought to be a consolidation of granulomas that mimics an alveolar process [3-5]. These opacities typically have a long and irregular shape and are more frequent in the perihilar region of the lungs [3,5]. Our patient’s CTA showed some of the typical sarcoidosis findings such as perihilar and mediastinal lymphadenopathy, as well as atypical findings such as consolidation in the right middle lobe.

Laboratory evaluation of sarcoidosis often reveals leukopenia, hypercalciuria, a slight elevation in serum alkaline phosphatase, hypergammaglobulinemia, an elevated erythrocyte sedimentation rate, and an elevated CRP [1,6]. Serological evaluation in patients with sarcoidosis often includes markers such as serum ACE (which is elevated in 75% of untreated patients) [1], adenosine deaminase, serum amyloid A, soluble interleukin-2 receptor, and D-dimer [1]. Our patient was found to have an elevated CRP and D-dimer while hospitalized; however, other common sarcoidosis markers were not found in this patient.

Our patient had some characteristics of worsening pneumonia as well as a few characteristics of sarcoidosis. Because the patient only had radiographic findings of pneumonia, no leukocytosis, and negative blood and BAL cultures, our patient likely had an acute exacerbation of sarcoidosis with typical and atypical pulmonological radiographic findings. However, the patient’s condition did improve with intravenous antibiotics, his CRP levels progressively declined, and his right middle lobe consolidation did improve, which points to a unique presentation of sarcoidosis.

There is no definitive diagnostic test for patients with sarcoidosis [1,6]. Therefore, the diagnosis consists of three elements that must be present for the diagnosis to be made. These elements include compatible clinical and radiographic manifestations, exclusion of other diseases that may present similarly, and histopathologic detection of noncaseating granulomas [1,6].

## Conclusions

In cases such as ours, it is important to take all findings into consideration and perform appropriate testing to help exclude other diseases that might present similarly to sarcoidosis. Our case was a unique presentation of sarcoidosis with the patient presenting with sepsis and only a few of the classical findings of sarcoidosis. Our patient had worsening pneumonia-like symptoms as well as airspace consolidation on imaging. This presentation is an example of how uncommon diseases such as sarcoidosis may present like common diseases such as pneumonia.
